# Endovascular Treatment of Abdominal Aortic Aneurysm: Impact of Diabetes on Endoleaks and Reintervention

**DOI:** 10.3390/jcm13123551

**Published:** 2024-06-17

**Authors:** Charlotte Praca, Natzi Sakalihasan, Jean-Olivier Defraigne, Nicos Labropoulos, Adelin Albert, Laurence Seidel, Lucia Musumeci

**Affiliations:** 1 Department of Cardiovascular and Thoracic Surgery, University Hospital of Liège, 4000 Liège, Belgium; charlotte.praca@chuliege.be (C.P.); nsaka@chuliege.be (N.S.); jo.defraigne@uliege.be (J.-O.D.); 2 Surgical Research Center, GIGA-Metabolism & Cardiovascular Biology Domain, University Hospital of Liège, 4000 Liège, Belgium; 3 Department of Surgery, Stony Brook University Hospital, Stony Brook, NY 11794-8191, USA; nicos.labropoulos@stonybrookmedicine.edu; 4 Biostatistics and Research Methods Center (B-STAT), University Hospital of Liège, 4000 Liège, Belgium; aalbert@uliege.be (A.A.); laurence.seidel@chuliege.be (L.S.)

**Keywords:** AAA, EVAR, diabetes, endoleaks

## Abstract

**Background:** Diabetes has a protective effect on abdominal aortic aneurysms (AAAs); however, there are contrasting reports on the impact of diabetes on endovascular aortic repair (EVAR) outcomes, endoleaks (ELs) being the major negative outcome. The present study characterizes ELs and their outcomes in AAA patients, diabetic or not. **Methods:** This single-center, retrospective, comparative study was carried out on 324 AAA patients who underwent elective EVARs between 2007 and 2016 at the University Hospital of Liège (Belgium). The primary endpoint was the incidence and effect of ELs on the evolution of the aneurysmal sac; the secondary endpoints were surgical reintervention and mortality rate. Diabetic and non-diabetic patients were compared with respect to various risk factors by logistic regression, while a Cox regression was used to analyze survival. **Results:** In AAA patients meeting the inclusion criteria (n = 248), 23% were diabetic. EL incidence was comparable (*p* = 0.74) in diabetic (38.7%) vs. non-diabetic (43.9%) patients. EL risk factors were age (HR = 1.04, *p* = 0.014) and fibrate intake (HR = 3.12, *p* = 0.043). A significant association was observed between ELs and aneurysm sac enlargement (*p* < 0.001), regardless of group (*p* = 0.46). Aneurysm sac regression per month for non-diabetic patients was −0.24 ± 0.013, while for diabetics it was −0.18 ± 0.027 (*p* = 0.059). Dyslipidemia (HR = 3.01, *p* = 0.0060) and sulfonylureas (HR = 8.43, *p* = 0.043) were associated with shorter EL duration, while diabetes (HR = 0.080, *p* = 0.038) and beta blockers (HR = 0.46, *p* = 0.036) were associated with longer EL duration. The likelihood of reoperation decreased with more recent surgery (OR = 0.90, *p* = 0.040), regardless of diabetic status. All-cause mortality was higher for the non-diabetic group (45.5% vs. 26.3%, *p* = 0.0096). **Conclusions:** Endoleak occurrence is a known risk factor for sac expansion. In diabetic patients, endoleaks lasted longer, and regression of the aneurysm sac tended to be slower. The number and type of reintervention was not related to the diabetic status of AAA patients, but overall survival was higher in patients with diabetes.

## 1. Introduction

Endovascular aortic repair (EVAR), introduced in 1991 by Parodi et al., has become the treatment of choice for patients with abdominal aortic aneurysm (AAA) [1]. In fact, EVARs reduce the risk of morbidity and mortality associated with open surgery. However, EVARs may lead to complications, such as the occurrence of endoleaks, which may result in aneurysm rupture. Endoleaks (ELs) are the most common and specific complication of endovascular treatment and are the primary cause of procedure failure. Endoleaks are defined as a persistent blood flow outside the stent graft into the aneurysm sac [2,3]. Even when an endograft is in place, the presence of endoleaks may cause sac expansion, potentially leading to the rupture of the aneurysm due to systemic pressure within the sac [4]. According to the classification described by Veith et al. [5] and later by Chaikof et al. [6] and Rokosh et al. [7], there are five types of endoleaks.

Several studies have focused on postoperative outcomes after EVARs [8], risk factors for endoleaks [2,3,4,9,10], and factors leading to surgical success [11,12,13,14]. A 3% annual failure rate for endovascular repair has been reported, with 1% due to rupture and 2% requiring conversion to open repair, compared to a failure rate of 0.3% for open repair. Up to 20% of patients experience stent complications within the first 5 years following an EVAR [2,3,4]. It has also been reported that long-term outcomes after endovascular repair are worse for large aneurysms, with most needing repair [15,16,17,18,19,20]. Frank Lederle was the first to draw attention to the beneficial effects of diabetes during the development and growth of AAAs [21,22,23]. Since his report, very few studies have focused on the impact of diabetes on endovascular treatment [24,25,26,27,28]. Some authors have concluded that diabetes has a protective effect after an EVAR, whereas others have shown that diabetes could be considered a risk factor for mortality and worse outcomes [24,25,26,27,28]. A meta-analysis published in 2017 involving 11,775 AAA patients did not find a correlation between occurrence of type II endoleaks and diabetes [8]. With this in mind, we conducted a retrospective study attempting to clarify the role of diabetes in EVAR outcomes (endoleaks, reinterventions, and mortality). More specifically, the objectives of this study were to determine the incidence of ELs and their characteristics and effects on aneurysm sac evolution in diabetic and non-diabetic patients, as well as the reintervention and mortality rates of the two groups.

## 2. Materials and Methods

### 2.1. Patient Population and Study Design

This was a retrospective, comparative study of 324 consecutive patients who had undergone an elective EVAR for an AAA at the University Hospital Center (CHU) of Liège (Belgium). Patients with a known diagnosis of syndromic and/or non-syndromic connective tissue disorders (e.g., Ehlers–Danlos, Marfan syndrome, and Loeys–Dietz syndrome) and patients who had undergone surgery for an infected and/or ruptured AAA were excluded. To be part of this study, patients also had to have undergone at least three control scans with contrast (one postoperative and another during the first year).

The study complied with the principles set out in the Declaration of Helsinki and is reported according to STROBE standards (http://www.strobe-statement.org/strobe-publications, accessed on 10 December 2016).

Patient demographic and imaging data were collected retrospectively from medical records and anonymized. Preoperative data included demographics, comorbidities, smoking, and treatments (beta-blockers, lipid-lowering agents, antiplatelet agents, anticoagulants, and diabetes medications). Perioperative variables included stent type or configuration. Postoperative data consisted of mortality, date of death, length of follow-up period, incidence of stroke or transient ischemic attack (TIA), acute myocardial infarction (AMI), peripheral vascular disease (PVD), endoleak, and reoperation. The size of the aneurysmal sac, its diameter (mm), was reported during the operation at 3 months, 6 months, 12 months, 18 months, 24 months, 36 months, 48 months, 60 months, 72 months, 84 months, 96 months, 108 months, and 120 months postoperatively, whenever such information was available. According to the requests of the Belgian healthcare system (INAMI), all patients underwent a CT scan at 3 months, at 1 year post EVAR, and each year for three years by CT examination, whereafter they were followed by CT or US (https://webappsa.riziv-inami.fgov.be/IRREQPublic/fr/Home/ListAllVersions/?code=161114, accessed on 10 December 2016). The maximum anteroposterior and transverse diameter was measured on the scanner by a single radiologist and two surgeons at each appointment.

### 2.2. Definitions

Study patients were classified as “diabetic” (confirmed by glycemic index) if they had suffered from diabetes for at least 10 years prior to the EVAR, or as “non-diabetic” if they had never received a diagnosis of diabetes.

The definitions of the five types of endoleaks, based on the classification of Veith [5], Chaikof [6], and Rokosh [7], are as follows: type I results from a lack of sealing between the proximal or distal edge of the stent and the native arterial wall, allowing blood to flow between them; type II, the most common type, occurs when blood flows into the aneurysm sac through the arterial branches (lumbar, inferior mesenteric artery, or other collateral vessels); type III results from a graft defect; type IV is due to porosity of the graft wall; and type V, also known as “endotension”, may be caused by the transmission of aortic blood pressure through the intraluminal graft/thrombus to the aneurysmal aortic wall, or by non-visible/unidentified leakage that cannot be detected by current imaging techniques. Endoleaks are also classified as “early endoleaks” or “late endoleaks” if they occur after EVAR interventions within 1 year or later, respectively.

Aneurysmal sac evolution was categorized as sac shrinkage, stability, and enlargement.

### 2.3. Statistical Analysis

Results were expressed as means and standard deviations (SD) for quantitative variables and as numbers (%) for categorical variables. Median and interquartile ranges (IQR) were used for durations. Groups (diabetic and non-diabetic) were compared using one-way analysis of variance or the non-parametric Kruskal–Wallis test for non-normal distributions. Normality was assessed using the Shapiro–Wilk test. Proportions were compared using chi-square and Fisher exact tests. Logistic regression and odds ratios (OR) with 95% confidence intervals (95%CI) were used to study the associations between binary outcome measures (e.g., endoleak occurrence) and several covariates, while Cox regression and hazard ratios (HR) were applied to time-to-event outcomes (e.g., overall survival, endoleak-free survival). Stepwise selection was used in the regression analyses to identify the most important variables as predictors. In particular, predictive capacity was assessed for each variable separately, and then to improve predictive capacity, other variables were added step by step. Entering and deleting significance levels were both set at 5%. The process was stopped when no additional variable contributed significantly to predictive capacity. To study and assess the potential effect of risk factors on the evolution of the aneurysm’s diameter, data were analyzed by general linear mixed-effects models, and results were expressed as regression coefficients with standard error (SE). Results were considered significant at the 5% critical level (*p* < 0.05). All *p*-values were given with two significant digits. Missing data were neither replaced nor imputed, and calculations were always performed using the maximum number of observations available. Statistical calculations were performed with SAS (version 9.4) (Cary, NC, USA) and R (version 4.1.0) (Wu Vienna) programs.

## 3. Results

### 3.1. Baseline Characteristics

Of the 324 patients who underwent EVARs, 248 AAA patients fulfilled the inclusion/exclusion criteria. Among the 57 patients classified as “diabetic”, one patient had type I and 56 type II diabetes. The two groups were similar in terms of sex and age, being predominantly males (97.2%) with a mean age of 73.4 ± 8.2 years (range: 53–94 years). Risk factors/comorbidity were equally prevalent in both groups, except for dyslipidemia affecting a higher percentage of diabetic patients compared to non-diabetic patients (Table 1).

### 3.2. Outcomes

The median (IQR) follow-up time for the population was 37.6 months (24.1–73.5 months), or a little over 3 years; non-diabetic patients were followed up with after a longer period compared to the diabetic group (Table 2). The median time between the EVAR and the last visit, i.e., the clinical examination follow-up, was 35.1 months (IQR: 16.3–59.3 months).

#### 3.2.1. Cardiovascular Events

Among the 248 EVAR patients, 6.5% experienced a stroke/TIA, 7.7% an AMI, and 18.3% a postoperative claudication. Outcome proportions were similar in both groups, except for PVD, which was observed more frequently in diabetic than in non-diabetic patients (28.1% vs. 15.3%, *p* = 0.029) (Table 2).

#### 3.2.2. Mortality

Overall mortality was 41.1% (102/248). Two deaths (2.0%) resulted from complications from the AAA intervention and all others from a reason unrelated to the intervention. All-cause mortality was significantly lower in diabetic than in non-diabetic patients (26.3% vs. 45.5%, *p* = 0.0092) (Table 2). Stepwise Cox regression applied to survival data showed that age was the only factor affecting mortality (HR = 1.08, 95%CI: 1.05–1.11, *p* < 0.0001). The median survival time post-surgery was 88.0 months (IQR: 44.2–133 months). After one year, the probability of survival was 98.8%, at 3 years 84.5%, and at 5 years 66.9%. The two EVAR-related deaths occurred at 27 months and 189 months post-surgery.

#### 3.2.3. Endoleaks

Endoleaks affected 39.9% of the whole population, and a similar proportion was found in diabetic and non-diabetic patients. Similarly, the two groups did not differ when comparing the type and duration of endoleaks (Table 2). Among patients with endoleaks, the majority had one endoleak (91 patients), 7 patients had 2, and one patient had 3, yielding a total of 108 endoleaks (Table 2). Among patients with more than 1 endoleak, 7 had at least one reintervention (88% against 19% in the group with 1 EL; *p* = 0.0002). On the other hand, there was no difference in sac enlargement or mortality between patients with 1 EL vs. >1 EL. Most patients were affected by type 2 endoleaks (83.3%). The majority (62%) of endoleaks were diagnosed within 3 months postoperatively. The median (IQR) time-lapse between EVAR and endoleak occurrence was 0.08 months (0–11.6 months) with 85 of the 108 endoleaks appearing early (<12 months) and 23 late (>12 months). No difference was seen between non-diabetic and diabetic patients (*p* = 0.93). The risk of at least one endoleak was similar in the diabetic and non-diabetic groups (38.7% vs. 43.9%, *p* = 0.74). Cox regression applied to time-to-endoleak occurrence showed that age (HR = 1.04, 95%CI: 1.01–1.07, *p* = 0.014) and fibrate consumption (HR = 3.12, 95%CI: 1.04–9.48, *p* = 0.043) were significant risk factors; after stepwise selection, only age remained significant (HR = 1.04, 95%CI: 1.01–1.06, *p* = 0.0071). Regarding the duration of endoleaks, Cox regression revealed that dyslipidemia (HR = 3.01, 95%CI: 1.37–6.61, *p* = 0.0060), and to a lesser extent sulfonylureas treatment (HR = 8.43, 95%CI: 1.08–66.2, *p* = 0.043), reduced duration, whereas diabetes (HR = 0.08, 95CI%: 0.01–0.87, *p* = 0.038) and beta-blockers (HR = 0.46, 95%CI: 0.23–0.95, *p* = 0.036) increased duration.

#### 3.2.4. Evolution of AAA Diameter

The evolution of the aneurysmal diameter after EVAR, as assessed by linear mixed-effects models, showed in the long term an average decline of 0.13 ± 0.0086 mm per month (*p* < 0.0001), with no difference between diabetic and non-diabetic patients (*p* = 0.16) (Supplementary Figure S1). A closer look at the graph, however, clearly shows a steeper drop in AAA diameters in the first years after EVAR. Thus, by restricting the evolution of AAA diameters to 60-months follow-up, the reduction in diameter remained highly significant (*p* < 0.0001), but the aneurysm sac regression tended to be larger in non-diabetic subjects than in diabetic patients (Table 2). After adjusting for diabetes status, a significant association was found between endoleak occurrence and an enlargement of the aneurysmal sac of 5 mm/year or more (OR = 11.6, 95%CI: 3.33–40.5, *p* < 0.0001). By contrast, there was no association between endoleak duration and aneurysmal sac enlargement, whether in non-diabetic (*p* = 0.61) or diabetic patients (*p* = 0.91). Globally, there were 78 early (<1 year post EVAR) endoleaks and 12 late (>1 year post EVAR) endoleaks, with similar proportions in non-diabetic and diabetic groups. The distribution of patients with early and late type 2 endoleaks differed significantly according to aneurysm sac evolution (shrinkage, stability, and enlargement), as described in Table 3.

Of the 39 patients with early type 2 endoleaks and aneurysm sac shrinkage, 12 were diabetic (60% of the diabetic patients with early type 2) and 27 were non-diabetic (46.5% of the non-diabetic patients with early type 2). On the other hand, 3 (75%) diabetic patients and 6 (75%) non-diabetic patients with late type 2 endoleaks (75%) evidenced aneurysmal sac stability.

#### 3.2.5. Secondary Interventions

Considering post-EVAR surgical outcomes, 28 (11.2%) of the 248 study patients underwent at least one secondary procedure (Table 2). The 28 reinterventions were embolization for 12 (42.9%) patients, stent placement for 8 (28.6%), and open approach for 8 (28.6%) subjects. Reintervention took place after a median time of 4.3 months (IQR: 0.10–16.6 months) after the start of endoleaks and after a median time of 29.0 months (IQR: 13.9–65.6 months) post EVAR. Considering each covariate separately, the risk of reintervention decreased with the time elapsed after EVAR (OR = 0.90, *p* = 0.040) and tended to be higher for patients with renal insufficiency (OR = 2.29, *p* = 0.066) and lower for beta-blocker users (OR = 0.45, *p* = 0.085). When combining all risk factors, only time after EVAR remained significant.

## 4. Discussion

The main objective of this retrospective, single-center, comparative study was to investigate, in patients with AAAs treated by EVARs, the impact of diabetic status on EL occurrence and outcome. Patients of the cohort were predominantly male (97.2%), aged 73.4 years, and active or past smokers (84.7%) with dyslipidemia (67.1%) and hypertension (75.7%), all well-known risk factors in the development of AAAs [29]. Since the discovery by Lederle of the protective effect of diabetes for AAAs [21], numerous studies have shown such a protective effect on the prevalence, growth, and rupture of AAAs, but have not elucidated the exact mechanism [11,21,22,23,29,30,31,32,33]. In this study, 23% of EVAR patients were diabetic (mean age 72.5 years), which is surprisingly close to the prevalence of diabetes in the general population aged ≥65 years in Belgium (21.2% in men and 15.3% among women) (https://atlas.ima-aim.be/jive?workspace_guid=5c20dcc6-14d4-4aff-87dd-4c9bda057dc2, accessed on 8 March 2024). We noticed that the proportion of diabetic versus non-diabetic patients treated with EVARs in our teaching hospital increased over the years, although the total number of EVAR procedures increased as well [8]. This is mainly due to the expansion of operative indications and the preference for EVAR treatment over open repair in diabetic patients, who often present more comorbidities [32]. Diabetic patients compared to non-diabetic patients had higher prevalence of dyslipidemia, which has long been known to be associated with diabetes, although the pathophysiology remains unclear [34,35].

The incidence of post-EVAR endoleaks ranged between 30% and 50% depending on the type of endoleak, with type 2 being the most common [36]. In agreement with previous studies [10,37,38], age was confirmed as a risk factor for endoleaks (HR =1.04, 95%CI: 1.007–1.069), while the impact of fibrate use (HR = 3.12, 95%CI: 1.04–9.38) was unclear, as stepwise selection showed no association, and the number of patients benefiting from such treatment was limited. Diabetes status did not affect EL incidence or incidence of type 2 ELs, confirming the results of Guo et al. [8]. Contrary to the findings of Png et al. [24], diabetes in our study did not appear to impact the growth of aneurysms or the need for reintervention after EVAR. It is well known that aneurysm sac growth after EVAR is most often the result of endoleaks [12,13], which was also demonstrated in our study (*p* < 0.0001). Additionally, as other studies have shown, diabetes tended to slow post-EVAR aneurysm sac shrinkage [10], which is an indicator of the success of endovascular treatment and is associated with low risk of postoperative complications [39]. Therefore, long-term follow-up after EVAR for diabetic patients would be necessary to achieve significant reductions in sac diameters. One hypothesis that could explain the slower healing process is the lower vascular compliance in diabetic patients due to the thickening of the vascular matrix and vascular calcification [33]. The median lifespan of endoleaks did not differ between diabetic patients and non-diabetic patients (60.7, 95%CI 12.4–60.7 months vs. 24.1, 95%CI 10.6–42.6 months, *p* = 0.30), although it was slightly higher in the former group. Lack of significance can be explained by a distorted median due to the size of our sample of diabetics and the number of endoleaks still active within the same sample of diabetics. However, accounting for this bias and looking at the average lifespan of endoleaks, Cox regression revealed increased duration of endoleaks in diabetic patients (persistent endoleaks) (HR = 0.08, 95%CI: 0.07–0.87, *p* = 0.038). It is known that persistent endoleaks are less likely to resolve spontaneously and increase postoperative risks [10], although in our study there was no association between EL duration and sac enlargement. Long-term follow-up should be recommended for individuals with late or persistent endoleaks. Reintervention should be performed only in cases of aneurysm sac enlargement and not in cases of persistent or late type 2 endoleaks with no sac growth. A large proportion of endoleaks lasted over 36 months post EVAR, both in diabetic and non-diabetic patients, but Belgian healthcare insurance covers the cost up to 3 years after EVAR [40,41] (INAMI—National Institute of Health and Disability Insurance in Belgium). It would therefore be appropriate to extend the monitoring of EVAR patients at least with ultrasound examinations.

No statistical difference was found between diabetics and non-diabetics when comparing the incidence of early or late type 2 endoleaks. As for the evolution of the aneurysm sac, although early type 2 endoleaks are associated with shrinkage of the aneurysm sac, no statistical difference was found in patients with type 2 endoleaks between diabetic and non-diabetic patients.

Early and late management of type 2 endoleaks is a recurring question among specialists practicing endovascular treatment of AAAs. According to our results, late type 2 endoleaks had a negative impact on aneurysm sac shrinkage, although we did not find a significant correlation between late type 2 endoleaks and aneurysm sac enlargement, unlike other results [39,41], probably due to the small size of the aneurysm sacs in our cohort. Nevertheless, patients with EVARs should be followed up with, as endoleaks can occur at any given time. Those with persistent and/or late endoleaks need to be studied further to determine the appropriate follow-up and intervention times. Interesting findings from a large retrospective study [42] demonstrated no survival benefit for those who had imaging follow-up compared to those who discontinued, indicating a selective approach on the follow-up of such patients. The criteria for such management should be developed from a prospective study. The frequency of secondary intervention in our study was 11%, while it was up to 22% in the ODYSSEOUS study [3,10,42,43,44]. The threshold for reintervention varies among centers based on the values given for the endoleak, the AAA, and patient characteristics. Here also, better definitions and criteria are needed to determine when and if an intervention should be performed. The chances of a reoperation were shown to significantly decrease over time (OR = 0.90), probably due to an improvement in the devices used and surgical techniques, but also due to a lack of long-term follow-up. Despite the absence of significant differences between the groups in the number and type of reinterventions, there was a trend towards embolization for diabetic patients (8.7% vs. 3.6%). Regarding the type of vessels embolized, the lumbar artery/middle sacral artery (57.1%) was more important than the inferior mesenteric artery (IMA) (28.6%) in non-diabetic patients. However, the proportion of lumbar artery/middle sacral artery (40%) and inferior mesenteric artery (40%) embolization was identical in the diabetic group. Given this information, and considering that endoleaks appear to last longer and require greater postoperative embolization, prophylactic IMA embolization could be suitable for diabetic patients and thus reduce the incidence of endoleaks [45].

More importantly, including in our study, it has not been shown that secondary intervention for type 2 endoleaks improves clinical outcomes. The ODYSSEOUS study, which was larger than ours, did not find any difference in the overall survival of patients with type 2 endoleaks and those without [43]. Furthermore, secondary intervention for the type 2 endoleaks did not have a survival impact compared to patients with conservative management. Together with our findings, this would indicate a conservative approach should be taken in patients with isolated type 2 endoleaks. However, all studies have been retrospective, with no direct and equal comparisons among patients with and without intervention. Therefore, a prospective study should be performed to provide robust data on the management of such patients.

Overall mortality was 41.1% (102/248) and the median survival time 88.0 months (IQR: 44.2–133 months) after surgery, while the median time between the last follow-up visit and death was 11.7 months (IQR: 5.1–31.7 months). After one year post-surgery, the probability of survival was 98.8%, at 3 years 84.5%, and at 5 years 66.9%. We noted that 2 patients (0.8%) died due to complications of the AAA (the first after 27 months and the second after 189.5 months), 40.3% died for other reasons, and 58.9% were alive at the end of follow-up. Multivariate analysis showed that age was the only risk factor affecting mortality (HR = 1.08, 95%CI: 1.05–1.11). Contrary to published series [26], all-cause mortality in this study was higher in the non-diabetic group than in the diabetic group (45.5% vs. 26.3%, *p* = 0.0096) but was the same if we compared the 3 categories of death (unrelated, linked, or not linked to the intervention). Note that the two patients who died following the intervention were non-diabetic. These results can be explained because of the strict monitoring of diabetic compared to non-diabetic patients, which is conducted occasionally by their general practitioners. More importantly, in a recent study of ours in patients with AAAs and >10 years follow-up, >90% of patients died from another reason not directly related to the AAA [46]. Although AAA-related mortality is still relevant, as most patients die for another reason, we may need to rethink our practices and take a different approach to AAA management.

## 5. Study Limitations

The retrospective nature of this study is a limitation, together with the absence of a control group. The limited number of patients in some of the subgroups may also hinder firmer conclusions. A multicenter study with an adequate number of patients would be more appropriate. Another limitation is that fact that we may have omitted variables that could influence the occurrence of endoleaks, such as preoperative anatomy, number of lumbar arteries and their patency, status of the inferior mesenteric artery, load in thrombus, presence of an associated iliac aneurysm, etc., and surgical variables such as stent extension.

## 6. Conclusions

This study focused on the role of diabetes on EVAR outcomes (endoleaks, reinterventions, and mortality) in AAA patients. Diabetes was identified as a risk factor for longer duration of endoleaks, but it did not appear to have an impact on their occurrence. Although aneurysm sac shrinkage in diabetic patients was slower compared to non-diabetic patients, there was no difference in sac enlargement. Additionally, diabetic patients were not subjected to more reinterventions compared to non-diabetic patients, and they had a longer survival time.

## Figures and Tables

**Table 1:** Baseline characteristic of AAA patients who underwent EVARs (N = 248) according to diabetic status. Proportions of each variable, indicated in parenthesis and expressed as a percentage, N (%), were compared between diabetic and non-diabetic groups. Differences are significant when *p*-value is <0.05.

Variable, N (%)	Diabetic N = 57 (23%)	Non-Diabetic N = 191 (77%)	*p*-Value
Sex (male)	56 (98.2)	185 (96.9)	0.99
Age (years)	72.1 ± 6.7	73.8 ± 8.6	0.16
Smoking history			0.73
Former	32 (56.1)	108 (56.8)	
Current	18 (31.6)	52 (27.4)	
HBP	44 (78.6)	143 (74.9)	0.57
COPD	24 (42.1)	62 (33.7)	0.25
RI	21 (36.8)	62 (33.0)	0.59
Dyslipidemia	46 (82.1)	119 (62.6)	0.0063
Stroke/TIA	10 (17.5)	19 (9.9)	0.12
AMI	28 (49.1)	72 (37.7)	0.12
PVD	7 (12.5)	16 (8.5)	0.36
Angina pectoris	12 (21.4)	35 (18.3)	0.60
Beta-blockers	31 (54.4)	92 (48.2)	0.41
Hypolipidemic drugs			0.21
Statins	38 (66.7)	102 (53.4)	
Fibrates	2 (3.5)	9 (4.7)	
Anti-platelets	51 (89.5)	180 (94.2)	0.23
Anti-coagulants	6 (10.5)	14 (7.3)	0.42
AAA diameter (mm)	59.4 ± 7.4	58.2 ± 8.2	0.32

HBP: hypertension, COPD: chronic obstructive pulmonary disease, RI: renal impairment, TIA: transient ischemic attack, AMI: acute myocardial infarction, PVD: peripheral vascular disease, AAA: abdominal aortic aneurysm, EVAR: endovascular aortic repair.

**Table 2:** Outcomes of AAA patients after EVARs (N = 248) according to diabetic status. Proportions of each variable were compared between diabetic and non-diabetic groups. Differences are significant when *p*-value is <0.05.

Variable, N (%)	Diabetic N = 57 (23%)	Non-Diabetic N = 191 (77%)	*p*-Value
Follow-up (months) *	27.9 (18.4–52.0)	39.7 (24.6–80.6)	0.013
Stroke/TIA	16 (6.5)	11 (5.8)	0.38
AMI	6 (10.5)	13 (6.8)	0.40
PVD	16 (28.1)	29 (15.3)	0.029
Death			0.0092
All-cause	15 (26.3)	87 (45.5)	
Intervention-related	0 (0.0)	2 (1.0)	
Endoleaks			
Total patients (N = 99)	25 (43.9)	74 (38.7)	0.74
Total endoleaks (N = 108)	27 (25)	81 (75)	0.12
Type 1	2 (7.4)	8 (9.9)	0.99
Type 1A	1 (3.7)	3 (3.7)	0.99
Type 1B	1 (3.7)	5 (6.2)	0.62
Type 2	24 (88.9)	66 (81.5)	0.35
Type 3	1 (3.7)	4 (4.9)	0.99
Type 4	0 (0.0)	2 (2.5)	0.99
EL duration (months) *	60.7 (12.4–60.7)	24.1 (10.6–42.6)	0.30
AAA sac enlargement (≥5 mm/year)	5 (8.8)	17 (8.9)	0.83
AAA sac regression, first 60 months (slope)	−0.18 ± 0.027	−0.24 ± 0.013	0.059
Reintervention			
Total	7 (12.3)	21 (10.9)	0.79
Embolization	5 (8.7)	7 (3.6)	
Stenting	1 (1.7)	7 (3.6)	
Open	1 (1.7)	7 (3.6)	

* Median (IQR); TIA: transient ischemic attack, AMI: acute myocardial infarction, PVD: peripheral vascular disease, AAA: abdominal aortic aneurysm, EVAR: endovascular aortic repair, IQR: interquartile range.

**Table 3:** Distribution of patients with early (<1 year post EVAR) and late (>1 year post EVAR) type 2 endoleaks according to aneurysm sac evolution (N = 90). Proportions (in parenthesis, n (%), calculated for total early or late type 2 ELs) of each sac evolution outcome were compared between early and late type 2 ELs groups. Differences are significant when *p*-value is < 0.05.

Aneurysm Sac Evolution	Early Type 2 ELs N = 78 (86.7%)	Late Type 2 ELs N = 12 (13.3%)	*p*-Value
Shrinkage	39 (50)	0 (0)	0.0048
Stability	28 (35)	9 (75)	
Enlargement	11 (15)	3 (25)	

## Data Availability

The data presented in this study are available on request from the corresponding author.

## Supplementary Materials

The following supporting information can be downloaded at https://www.mdpi.com/article/10.3390/jcm13123551/s1: Figure S1: Variation of aneurysmal diameter in diabetic versus non-diabetic patients.
