# Supplementary material for: Endovascular Treatment of Abdominal Aortic Aneurysm: Impact of Diabetes on Endoleaks and Reintervention
Source: J Clin Med. 2024 Jun 17;13(12):3551. doi: 10.3390/jcm13123551 (PMC11204582; doi:10.3390/jcm13123551)
Supplement: Supplementary file 1 [file jcm-13-03551-s001.zip › jcm-3044545-supplementary.docx]

**Supplemental data**

**Figure S1.** Variation of aneurysmal diameter in diabetic versus nondiabetic patients.
